# Advances in Vaccines Against Infectious Diseases

**DOI:** 10.3390/vaccines14090796

**Published:** 2026-09-10

**Authors:** Salvatore Giovanni De-Simone

**Affiliations:** Centro de Desenvolvimento Tecnológico em Saúde [CDTS], Fundação Oswaldo Cruz [FIOCRUZ], Rio de Janeiro 21040-900, Brazil; salvatore.simone@fiocruz.br

## 1. Introduction

### 1.1. Advances in Vaccines Against Infectious Diseases

Vaccination remains one of the most successful interventions in medical history. Over the past two centuries, vaccines have transformed the epidemiology of infectious diseases, substantially reducing morbidity, mortality, disability, and healthcare costs. The global impact of immunization is particularly evident in the achievements of the EPI, established by the WHO in 1974. A recent modeling analysis estimated that vaccination has averted approximately 154 million deaths since the establishment of the EPI, including 146 million deaths among children younger than five years, and has contributed substantially to the global reduction in infant mortality [1]. Nevertheless, the benefits of vaccination remain unevenly distributed. The WHO Immunization Agenda 2030 emphasizes that immunization must reach all individuals, at every stage of life, while addressing persistent inequities in vaccine access, coverage, surveillance, and health system capacity [2,3,4].

At the same time, infectious diseases are continuing to evolve. Antigenic variation, immune evasion, AMR, zoonotic emergence, climate change, population mobility, and ecological disruption create an increasingly complex infectious disease landscape. Pathogens for which effective vaccines remain unavailable or incompletely effective, including HIV, tuberculosis, malaria, many bacterial pathogens, and several emerging viruses, illustrate the limitations of traditional vaccine development strategies. Therefore, the central challenge of modern vaccinology is no longer simply producing vaccines, but rationally designing vaccines that generate durable, broad, appropriately localized, and protective immunity against pathogens with complex biology [5,6].

The field is consequently undergoing a profound technological transformation. Advances in genomics, transcriptomics, structural biology, immunoinformatics, artificial intelligence, high-throughput screening, protein engineering, nanotechnology, and nucleic acid delivery are redefining how researchers discover, optimize, formulate, and evaluate antigens. The COVID-19 pandemic powerfully demonstrated what can be achieved when these technologies are integrated with established vaccine platforms. However, the next generation of vaccines must go beyond rapid development against a single emerging pathogen. It should address antigenic diversity, mucosal transmission, immune imprinting, immunosenescence, pathogen persistence, and the need for broad protection against pathogen families.

### 1.2. From Conventional Vaccinology to Precision Vaccine Design

Classical vaccines based on live attenuated or inactivated microorganisms remain essential components of global immunization programs. Protein subunit, toxoid, polysaccharide, conjugate, and VLP vaccines have further expanded the range of available vaccine technologies. However, conventional approaches can be constrained by pathogen cultivation, manufacturing complexity, antigenic variability, safety considerations, and the difficulty of identifying the components that induce protective immunity.

The introduction of reverse vaccinology represented a major conceptual transition. Rather than beginning with pathogen cultivation and empirical antigen purification, reverse vaccinology uses genome sequences and computational analyses to systematically identify candidate antigens [4,5,7]. The successful development of vaccines against *Neisseria meningitidis* serogroup B provided compelling validation of reverse vaccinology’s practical value. By combining genome-derived antigen discovery with immunological and functional screening, researchers developed multicomponent vaccines that address substantial antigenic diversity [8]. This paradigm has since contributed to a broader transformation in vaccine development, supporting strategies to address antimicrobial resistance, strengthen pandemic preparedness, and promote more equitable access to effective vaccines [9,10,11,12].

Today, reverse vaccinology is increasingly being complemented by “reverse vaccinology 2.0”, structural vaccinology, immunopeptidomics, and systems vaccinology. High-resolution structural information can reveal conserved neutralizing epitopes and guide antigen stabilization in immunologically favorable conformations. The development of respiratory syncytial virus (RSV) vaccines illustrates this principle particularly well. Structural studies of the prefusion F glycoprotein identified highly vulnerable epitopes, allowing rational stabilization of the prefusion antigen and directly contributing to the development of effective RSV vaccines [8,13].

This paradigm is especially important for pathogens in which the dominant immune response targets variable or non-protective regions. Rather than simply reproducing a pathogen antigen, future vaccines can be engineered to focus immune responses on conserved functional regions [14].

### 1.3. Emerging Vaccine Platforms: Vectors and RNA Technologies

Perhaps the most visible transformation in vaccinology has been the emergence of platform technologies, particularly viral vectors and nucleic acid vaccines. Each platform has distinct strengths and limitations, and its optimal implementation depends on the pathogen, target population, desired immune response, and practical vaccination requirements. Replicating and non-replicating viral vectors can efficiently deliver antigens and induce robust cellular and humoral immunity, but pre-existing vector immunity, vector-specific responses, manufacturing complexity, and safety considerations can limit their application. Replicating vectors may be inappropriate for some individuals with significant immune deficiencies, emphasizing that platform selection should follow a fit-for-purpose framework rather than the assumption that a single technology is universally optimal.

The COVID-19 pandemic established mRNA vaccines as a transformative platform, demonstrating that nucleic acid technologies can combine rapid antigen design, scalable manufacturing, and adaptability to viral evolution [15,16,17]. Their potential extends well beyond SARS-CoV-2, and researchers are investigating mRNA approaches against diverse viral, bacterial, and parasitic pathogens. Major advantages include rapid modification of antigen sequences, the ability to encode complex or multiple antigens, and cell-free manufacturing without cultivating large quantities of infectious organisms. Lipid nanoparticles are equally important, as they protect RNA, facilitate cellular uptake and endosomal escape, and influence the magnitude and quality of the resulting immune response [17,18,19].

However, mRNA technology should not be regarded as an “ultimate fit-for-all” solution. Important knowledge gaps remain concerning intracellular trafficking, tissue distribution, antigen production and clearance, and the determinants governing the magnitude and duration of antigen expression following vaccination [17,19]. Understanding these kinetics is particularly important for optimizing dose, formulation, route, and immunization schedule. In addition, rare but potentially serious adverse events, including myocarditis after receiving some mRNA COVID-19 vaccines, require continued investigation of their underlying biological mechanisms [20].

Other unresolved questions concern the quality and durability of antibody responses. Repeated mRNA vaccination can alter the IgG subclass profile, including increased antigen-specific IgG4 responses, although the functional and clinical implications of this shift remain incompletely understood [21]. Moreover, recent evidence indicates that mRNA vaccination poorly establishes SARS-CoV-2-specific antibody-secreting cells in the bone marrow, a long-lived plasma cell compartment, which may contribute to the relatively rapid decline in circulating antibodies [22].

Reactogenicity and implementation feasibility also require consideration. Although generally transient, systemic adverse reactions following some mRNA vaccines can affect their acceptability and complicate implementation in settings where short-term absenteeism has important operational consequences, such as healthcare, emergency services, and security personnel. Similarly, unexpected clinical observations in pediatric trials of respiratory virus vaccines should be carefully investigated rather than automatically attributed to the platform. These examples highlight the importance of evaluating novel vaccines not only by immunogenicity, but also by clinical outcomes, durability, safety, tolerability, and population-specific performance.

The next generation of RNA vaccines is likely to include self-amplifying, trans-amplifying, circular RNA, and other emerging modalities that may increase antigen expression while reducing the amount of RNA required per dose [19,23]. Nevertheless, these technologies introduce additional questions concerning control of antigen expression, innate immune activation, tissue targeting, stability, manufacturing consistency, and long-term safety. The future of RNA vaccination will therefore depend not only on optimizing the RNA molecule but on rationally engineering the entire vaccine system, including antigen sequence, RNA chemistry, untranslated regions, delivery vehicle, route of administration, adjuvant activity, dose, and schedule.

Ultimately, emerging vaccine platforms should be evaluated according to a fit-for-purpose principle. Viral vectors, RNA technologies, protein-based vaccines, nanoparticles, and other platforms should be selected based on the pathogen, the desired immune response, the characteristics of the target population, and the practical requirements of vaccination. Rather than searching for a universal vaccine technology, precision vaccinology should seek the optimal platform–antigen–adjuvant–delivery combination for each pathogen and target population.

### 1.4. Nanoparticles, Virus-like Particles, and Multivalent Antigen Presentation

Nanotechnology offers another major opportunity to improve vaccine immunogenicity. Nanoparticles can mimic aspects of pathogen architecture, increase antigen stability, facilitate lymph-node delivery, and promote efficient interaction with antigen-presenting cells. Researchers are investigating protein nanoparticles, VLPs, lipid nanoparticles, polymeric particles, and other self-assembling structures as platforms for presenting antigens in highly ordered, repetitive configurations [24,25].

The repetitive arrangement of antigens on nanoparticles can efficiently cross-link B-cell receptors and enhance germinal-center responses. More importantly, nanoparticles can be engineered to display multiple antigens or conserved epitopes simultaneously. This property is particularly attractive for pathogens characterized by extensive antigenic variation.

Multivalent and mosaic vaccine strategies are becoming increasingly important for influenza, coronaviruses, HIV, and other rapidly evolving pathogens characterized by substantial antigenic diversity. Rather than relying primarily on predictions of the next dominant strain, these approaches aim to broaden immune recognition by presenting conserved epitopes or antigenic regions derived from multiple strains, variants, or pathogen lineages. By reducing dependence on a single variable antigen, these strategies can enhance the breadth and durability of protective immunity and may offer greater resilience to antigenic evolution and immune escape.

### 1.5. Epitope-Based and Multiepitope Vaccines

Identifying B- and T-cell epitopes represents another important frontier in precision vaccinology. Peptide-based approaches allow the selection of conserved immunogenic regions while avoiding irrelevant, variable, or potentially undesirable portions of pathogen proteins.

High-throughput peptide synthesis and peptide arrays, including SPOT synthesis, have enabled systematic mapping of antibody recognition across entire proteins. Such approaches can identify linear B-cell epitopes recognized by IgM, IgG, IgA, or other antibody classes and can reveal differences in immune recognition associated with disease stage, exposure history, vaccination, or pathogen subtype [26].

Importantly, epitope mapping should not be regarded solely as a diagnostic strategy, as it can also provide a rational foundation for vaccine design. Conserved epitopes identified through immunological screening can be further characterized using sequence alignment (BLAST, Basic Local Alignment Search Tool), structural modeling, immunoinformatics, and population coverage predictions to assess their conservation, accessibility, and potential immunogenicity. Researchers can then incorporate promising epitopes into multiepitope vaccine constructs, including recombinant chimeric proteins, synthetic peptides, virus-like particles (VLPs), nanoparticles, and nucleic acid-based platforms.

A major advantage of multiepitope vaccination is the ability to simultaneously induce complementary immune responses. B-cell epitopes can promote antibody-mediated neutralization or opsonization, whereas CD4+ and CD8+ T-cell epitopes may support cellular immunity. Combining conserved epitopes from different antigens may also reduce the likelihood that pathogen variation will completely escape vaccine-induced immunity.

Nevertheless, epitope selection requires careful consideration. Immunogenicity does not necessarily imply protection. Antibody binding should ideally be complemented by functional assays, such as neutralization, opsonophagocytosis, bactericidal activity, inhibition of adhesion or invasion, or other pathogen-specific functional measurements. Similarly, computational prediction of T-cell epitopes requires experimental confirmation. Future epitope-based vaccines should therefore integrate high-throughput screening with structural and functional validation.

### 1.6. Novel Adjuvants and Precision Vaccinology

A major challenge in vaccinology is that measured immune responses do not always translate into clinical protection. Systems vaccinology integrates molecular, cellular, and immunological data to identify signatures associated with effective or inadequate vaccine responses. Transcriptomics, proteomics, metabolomics, single-cell sequencing, immune-repertoire analysis, and high-dimensional cytometry can reveal biomarkers and mechanistic pathways underlying poor responsiveness, providing opportunities for both risk stratification and targeted intervention.

Novel adjuvants represent an important strategy for improving vaccine responses. Rather than simply increasing antibody production, adjuvants can shape the magnitude, quality, breadth, durability, and localization of humoral and cellular immunity. Advances in innate immunology have enabled more rational activation of pathways involved in antigen presentation, germinal center formation, antibody maturation, and B- and T-cell memory. Importantly, next-generation adjuvants should not only aim to induce stronger immunity, but also to promote robust, finely tailored cellular responses, including appropriately polarized CD4^+^ and CD8^+^ T-cell responses, according to the pathogen’s biological requirements.

This is particularly relevant for older adults, infants, immunocompromised individuals, and other groups with heterogeneous immune responsiveness. Systems-level analyses may identify the mechanisms behind inadequate responses and guide the selection of adjuvants, antigens, delivery platforms, or immunization schedules that overcome specific immunological barriers.

Thus, systems vaccinology and adjuvant innovation represent complementary components of precision vaccination: systems vaccinology can identify which individuals respond poorly and why, while mechanistic insights can guide interventions that optimize both humoral and cellular immunity. The future of adjuvantology therefore lies in programming the right immune response in the right population, generating robust, durable, and appropriately tailored cellular and antibody responses rather than indiscriminately maximizing immune activation.

### 1.7. Mucosal Immunity: Bringing Vaccines to the Portal of Infection

Most infectious pathogens enter the human body through mucosal surfaces. Respiratory, gastrointestinal, and genitourinary mucosa therefore represent critical sites for preventing infection and transmission. Yet most currently licensed vaccines are administered intramuscularly and primarily generate systemic immune responses.

Consequently, developing mucosal vaccines is an important priority. Local production of secretory IgA, tissue-resident memory T cells, and other components of mucosal immunity could provide a first line of defense at the site where pathogens initially encounter the host [27,28].

Researchers are investigating intranasal, oral, inhaled, and other mucosal delivery systems using live vectors, protein antigens, VLPs, nanoparticles, and nucleic acid formulations. However, mucosal vaccination presents unique challenges, including antigen degradation, limited residence time, tolerogenic mechanisms, anatomical barriers, and the need to achieve sufficient immunogenicity without excessive inflammation [29,30,31].

Developing effective mucosal vaccines may be particularly important for respiratory viruses. Sterilizing or infection-blocking immunity could potentially reduce not only severe disease but also transmission. Thus, vaccine efficacy should increasingly be considered at multiple levels: preventing infection, reducing disease severity, preventing transmission, and reducing long-term complications.

Mucosal adjuvants are an important tool for improving vaccine efficacy, particularly when conventional formulations fail to induce sufficiently strong or durable immunity. By modulating innate immune pathways and antigen presentation, novel adjuvants can enhance and fine-tune both humoral and cellular responses. Examples include TLR agonists, cytokine-based adjuvants, saponins, bacterial-derived molecules, and nanoparticle-based systems, which can promote distinct immune profiles according to the pathogen and target population. Rational adjuvant selection can generate robust, fine-tailored cellular and mucosal responses while reducing antigen requirements and improving the durability and breadth of protection. Thus, next-generation adjuvants represent a critical component of precision vaccine design, particularly for pathogens for which traditional vaccine approaches have provided limited protection [32,33,34,35,36].

### 1.8. Tackling Pathogens That Remain Difficult Vaccine Targets

Despite extraordinary progress, vaccination still inadequately controls several major infectious diseases.

#### 1.8.1. HIV

HIV remains one of the most difficult vaccine targets because of its extraordinary genetic diversity, extensive glycan shielding, rapid evolution, and ability to establish persistent infection. Conventional vaccine approaches have struggled to induce broadly neutralizing antibodies (bnAbs).

Structure-guided and germline-targeting strategies represent a fundamentally different approach. These vaccines aim to activate rare naive B-cell precursors that can develop into bnAb-producing lineages through sequential immunization. Recent clinical studies have provided evidence that rationally designed germline-targeting immunogens can activate and guide VRC01-class B-cell responses in humans [37,38]. Although a protective HIV vaccine remains an unresolved challenge, these studies demonstrate the increasing ability of vaccinologists to manipulate specific B-cell developmental pathways.

#### 1.8.2. Tuberculosis

Tuberculosis remains another major challenge. BCG provides important protection against severe childhood tuberculosis but has variable efficacy against pulmonary disease in adults. Improved tuberculosis vaccines are therefore a major global priority.

New approaches include recombinant protein vaccines, viral vectors, improved BCG formulations, novel adjuvants, and vaccines designed to enhance T-cell responses. Continued investment in tuberculosis vaccine research is particularly important for high-burden countries, including Brazil, where tuberculosis remains a major public health problem [16].

#### 1.8.3. Malaria

The recent development of malaria vaccines represents a historic milestone. RTS, S/AS01 and R21/Matrix-M target the circumsporozoite protein during the pre-erythrocytic stage of *Plasmodium falciparum* infection. Their development demonstrates that effective vaccination against a complex eukaryotic parasite is possible, although protection remains incomplete and vaccination must be integrated with vector control, surveillance, diagnosis, and treatment [39,40].

Future malaria vaccines may need to combine antigens from multiple parasite life-cycle stages and induce stronger, more durable immunity.

#### 1.8.4. Influenza and Rapidly Evolving Respiratory Viruses

Influenza illustrates the persistent challenge posed by antigenic evolution [41]. Current seasonal vaccines provide important protection but require periodic reformulation because of antigenic drift. Universal influenza vaccine strategies aim to induce broader and longer-lasting immunity against conserved viral components and could substantially reduce the need for frequent vaccine updates and annual boosting [31,42]. However, broad antigenic coverage alone will not solve all of the challenges associated with influenza vaccination. Even a highly conserved and broadly protective antigen may fail to elicit adequate immunity in individuals with impaired vaccine responsiveness. This is particularly relevant in older adults in whom immunosenescence, chronic inflammation, altered B- and T-cell function, previous exposure to influenza viruses and vaccines, and other host-specific factors can substantially influence vaccine responses [43,44,45].

Importantly, poor responsiveness is not a uniform feature of aging. Rather, older adults display considerable heterogeneity in their capacity to mount effective vaccine-induced immune responses, with a clinically relevant subset showing particularly limited responses despite repeated vaccination [45,46]. Understanding the biological mechanisms underlying this heterogeneity should therefore become an important objective of next-generation influenza vaccinology. Systems vaccinology, immunophenotyping, and longitudinal analyses may help identify molecular and cellular signatures associated with poor responsiveness and distinguish individuals who require alternative vaccination strategies from those who respond adequately to conventional approaches [46,47]. Such knowledge could enable tailored interventions for low-responder populations, including optimized antigens, more appropriate adjuvants, higher antigen doses, alternative delivery platforms, mucosal or T-cell-focused approaches, and potentially individualized vaccination schedules.

This distinction between antigenic breadth and host responsiveness has broader implications for vaccine development. The goal of a universal influenza vaccine should not simply be to create an antigen that recognizes a wider range of viral strains, but also to generate effective and durable immunity across heterogeneous human populations. Thus, future influenza vaccines may need to combine broad-spectrum antigen design with strategies that actively overcome or compensate for host-specific limitations in immune responsiveness.

Similar challenges have emerged with SARS-CoV-2, where rapid variant evolution and immune escape have highlighted the limitations of vaccines focused predominantly on variable immunodominant regions [45,48]. Increasingly, vaccine strategies therefore target conserved epitopes and aim to protect against multiple variants or, ultimately, multiple members of a viral family. Structure-guided antigen design, immunofocusing, nanoparticle display, multivalent and mosaic antigen presentation, and rational selection of conserved targets represent promising approaches for expanding the breadth of immunity [48,49,50]. As with influenza, however, broader antigenic coverage should be viewed as one component of a broader strategy. Next-generation respiratory virus vaccines will likely need to integrate antigenic breadth, durability, appropriate tissue localization of immunity, and the biological determinants of individual vaccine responsiveness. Such an approach would move respiratory virus vaccinology beyond the paradigm of simply matching viral evolution toward precision vaccination tailored to both pathogen diversity and host immune heterogeneity.

### 1.9. Vaccines as an Instrument Against Antimicrobial Resistance

Vaccination is increasingly recognized as an important component of the global response to AMR. Preventing bacterial infections directly reduces the need for antibiotic treatment and therefore decreases selective pressure favoring resistant organisms [9,10,43].

The potential impact extends beyond currently licensed vaccines. Pneumococcal, *Haemophilus influenzae* type b, influenza, and other vaccines show that preventing infection can indirectly reduce antimicrobial use. New vaccines targeting pathogens such as *Staphylococcus aureus*, *Klebsiella pneumoniae*, *Clostridioides difficile*, antimicrobial-resistant enteric bacteria, and other healthcare-associated pathogens could become important components of future AMR control strategies.

However, vaccine development against bacterial pathogens is often difficult because protective immunity may depend on complex interactions among antibodies, phagocytes, mucosal immunity, and cellular responses. The pathogen may also display extensive antigenic diversity. Consequently, reverse vaccinology, pan-genomic analyses, proteomics, immunopeptidomics, and machine-learning approaches may be particularly valuable for identifying conserved protective targets.

Vaccines should therefore be considered not merely as preventive health interventions but as part of a broader antimicrobial stewardship strategy.

### 1.10. Artificial Intelligence and Computational Vaccinology

Artificial intelligence and machine learning are rapidly becoming integral to vaccine research. Large genomic and proteomic datasets can be analyzed to identify conserved regions, predict antigenicity, characterize immune epitopes, model protein structures, and prioritize vaccine candidates.

Integrating AI with structural biology has particular potential. Protein structure prediction and molecular modeling can help identify accessible epitopes, predict antigen conformations, and design stabilized proteins. Machine-learning algorithms can also help predict HLA binding, population coverage, antibody recognition, and potential antigenic evolution.

However, AI should not replace experimental immunology. A predicted epitope is not necessarily immunogenic, and an immunogenic epitope is not necessarily protective. The greatest potential lies in integrating computational prioritization with experimental validation. The future workflow may increasingly resemble a continuous cycle of genomic surveillance, computational antigen design, high-throughput screening, structural validation, and rapid clinical translation.

### 1.11. Systems Vaccinology and the Search for Correlates of Protection

A major challenge in vaccinology is that measured immune responses do not always translate directly into clinical protection. Antibody titers, cellular responses, and other conventional immunological measurements provide valuable information, but they may not fully capture the complex biological processes that determine whether an individual develops effective and durable protection. Systems vaccinology offers a powerful framework for addressing pathogen characteristics and the biological determinants of individual- and population-level immune responsiveness.

### 1.12. Beyond Efficacy: Durability, Breadth, and Quality of Immunity

Evaluating future vaccines should move beyond simple measurements of antibody titers. A vaccine that induces high antibody concentrations for a few months may be less valuable than one that generates moderate but durable and functionally broad immunity.

Important dimensions include antibody affinity, neutralization breadth, Fc-mediated functions, memory B cells, long-lived plasma cells, CD4+ and CD8+ T-cell responses, tissue-resident immunity, and mucosal immune responses.

Distinguishing immunogenicity from protection is particularly important. Vaccine-induced antibodies may recognize an antigen without preventing infection, while cellular immunity may substantially reduce disease severity. Therefore, clinical development programs should include mechanistic studies that identify correlates of protection and distinguish immune responses that are merely measurable from those that are functionally meaningful.

### 1.13. Pandemic Preparedness and Platform Technologies

The COVID-19 pandemic demonstrated that vaccine platforms can dramatically accelerate responses to emerging pathogens. The next stage of pandemic preparedness [51] should therefore focus on platform technologies that can be rapidly adapted to new pathogens, rather than developing every vaccine from scratch.

Plug-and-play platforms based on mRNA, self-amplifying RNA, viral vectors, recombinant proteins, and nanoparticles can retain manufacturing and regulatory knowledge while replacing the antigenic component. This approach may substantially reduce the time between pathogen identification and clinical evaluation [52].

Preparedness should also include genomic surveillance, biobanking, standardized assays, regulatory harmonization, manufacturing capacity, clinical trial networks, and equitable vaccine distribution mechanisms. Technology alone will not prevent future pandemics if manufacturing and access remain concentrated in a small number of countries.

### 1.14. Equity, Accessibility, and the Future of Global Vaccination

Scientific innovation must be accompanied by equitable access. The history of vaccination shows that a vaccine’s public health impact depends not only on efficacy but also on whether it can be manufactured, distributed, stored, administered, and accepted at a large scale.

Thermostability, simplified dosing schedules, low-cost manufacturing, needle-free administration, and decentralized production may therefore become as important as antigen design. Technologies that require sophisticated cold chains or highly specialized manufacturing can have limited impact on low-resource settings.

The next generation of vaccines should thus be designed with global accessibility in mind from the earliest stages of development. This includes consideration of dose-sparing strategies, thermostable formulations, mucosal delivery, regional manufacturing capacity, technology transfer, and sustainable financing.

The WHO IA2030 framework emphasizes that immunization is an essential component of primary healthcare and global health security [2,3]. Recent assessments indicate that many IA2030 targets remain off track, reinforcing the need to combine scientific innovation with stronger immunization systems and renewed efforts to reach underserved populations [3].

### 1.15. One Health and Vaccines Against Emerging Infections

Many emerging infectious diseases arise at the interface of humans, animals, and the environment. A One Health perspective is therefore essential in future vaccine development.

Vaccines for livestock and wildlife can reduce pathogen circulation and zoonotic transmission, while human vaccination can protect populations exposed to emerging pathogens. Climate change and environmental disruption may alter vector distributions and create new opportunities for pathogen emergence.

Vaccine development should therefore increasingly incorporate veterinary epidemiology, genomic surveillance, ecological modeling, and environmental monitoring. Platform technologies could become particularly valuable when pathogens emerge unexpectedly in animal reservoirs and subsequently cross species barriers.

### 1.16. The Road Ahead

The future of vaccinology will not be defined by a single platform. Instead, it will emerge from the convergence of multiple technologies.

The vaccines of the coming decade are likely to combine rational antigen design, genomic surveillance, AI-assisted epitope prediction, structural biology, multiepitope engineering, nanoparticles, nucleic acid platforms, optimized adjuvants, and mucosal delivery. Rather than relying exclusively on empirical trial-and-error approaches, vaccine development will increasingly become a data-driven engineering discipline.

Several priorities should guide this transition. First, conserved, functionally relevant epitopes should be identified and experimentally validated. Second, vaccine formulations should be designed to induce the appropriate type of immunity for the pathogen and anatomical site of infection. Third, vaccine development should address durability and breadth rather than focusing exclusively on short-term antibody responses. Fourth, vaccine development priorities should explicitly consider AMR. Fifth, platform technologies should be integrated into pandemic preparedness strategies. Finally, technological innovation must be accompanied by equitable access and sustainable immunization systems.

The field has already transitioned from empirical vaccinology toward rational vaccine design. The next step is precision vaccinology: deliberately selecting antigens, epitopes, delivery systems, adjuvants, routes, and schedules to generate the precise immune response required for protection.

## 2. Conclusions

Vaccines remain among the most powerful tools available for preventing infectious diseases, and their historical contribution to global health is unequivocal. Yet the infectious disease threats of the twenty-first century require new solutions. Antigenic variation, pathogen immune evasion, antimicrobial resistance, zoonotic emergence, and persistent inequities in vaccine access demand a new generation of vaccines that are broader, more durable, and adaptable, and easier to deploy.

The convergence of reverse vaccinology, structural vaccinology, epitope mapping, systems immunology, artificial intelligence, mRNA and other nucleic acid platforms, nanoparticles, VLPs, mucosal delivery, and precision adjuvantation provides an unprecedented opportunity to achieve these goals.

Importantly, innovation should not replace conventional vaccination programs. Rather, new technologies should complement the highly effective vaccines already available while addressing diseases for which current vaccines are inadequate or nonexistent.

The future of infectious disease prevention will depend on our ability to translate biological complexity into rational vaccine design. This is a substantial challenge, but scientific foundations are stronger than ever. Advances in vaccinology now offer the possibility of responding more rapidly to emerging infections and tackling some of the oldest and most persistent infectious diseases that continue to affect global health.

For these reasons, continued interdisciplinary research spanning microbiology, immunology, structural biology, genomics, bioinformatics, nanotechnology, biotechnology, epidemiology, clinical medicine, and public health will be essential. The next generation of vaccines should ultimately be judged not only by their immunogenicity or efficacy in clinical trials, but also by their capacity to provide durable protection, reduce transmission, combat antimicrobial resistance, prevent future outbreaks, and reach populations that need them most.

## Abbreviations

The following abbreviations are used in this manuscript:

EPI	Expanded Program on Immunization
WHO	World Health Organization
IA2030	Immunization Agenda 2030
AMR	Antimicrobial resistance
VLP	Virus-like particle
RSV	Respiratory syncytial virus
bnAbs	Broadly neutralizing antibodies
BCG	Bacillus Calmette–Guérin
HIV	Human immunodeficiency virus
COVID-19	Coronavirus disease 2019
SARS-CoV-2	Severe acute respiratory syndrome coronavirus 2
